# Assessment of Outcome in Hypospadias Surgery – A Review

**DOI:** 10.3389/fped.2014.00002

**Published:** 2014-01-20

**Authors:** Alexander Springer

**Affiliations:** ^1^Department of Pediatric Surgery, Medical University of Vienna, Vienna, Austria

**Keywords:** hypospadias, complications, assessment, outcome, audit

## Abstract

Hypospadias is a challenging field of urogenital reconstructive surgery with different techniques being currently used. Modern surgery claims that it is possible to create a functionally and cosmetically normal penis. Continuous re-evaluation and assessment of outcome may have a major impact on future clinical practice. Assessment of outcome includes: complication rate, cosmetic appearance of the penis, functional outcome (micturition, sexuality), and psychological factors such as quality of life and psychosexual life. This article briefly reviews current strategies of outcome assessment. Somehow in the future, we will be able to give an accurate estimation of the long-term consequences of being born with hypospadias.

## Introduction

Traditionally, successful repair of hypospadias was defined as straight penis in erection and a meatus near the tip of the glans, permitting voiding in a standing position and allowing sexual intercourse. Nevertheless, modern surgery claims that it is possible to create a functionally and cosmetically normal penis. Myriads of techniques have been described and there is still evolution going on. The majority of publications present single-center and single-surgeon retrospective case series with a limited follow-up period and a limited number of patients undergoing follow-up. High-quality randomized trials in pediatric urology are extremely challenging and therefore rarely performed (1). Case series reporting reliable and valid data should include inclusion and exclusion criteria, a detailed description of the surgical procedure, study design, primary and secondary outcome parameters, and follow-up period and percentage of patients undergoing follow-up (2). There are some systematic reviews and meta-analysis comparing different techniques (3–5). These reviews criticize that there are no standardized algorithms for assessment of outcome. Comparison of studies therefore is complicated, if not impossible. From the clinical point of view, continuous assessment of outcome represents quality control and is part of clinical governance. Continuous re-evaluation may have a major impact on future clinical practice.

Assessment of outcome includes:

- Complications
- Cosmetic appearance of penis
- Functional outcome (micturition, sexuality)
- Quality of life and psychosexual life.

## Complications

The most common complications following hypospadias repair are: urethrocutaneous fistula, meatal stenosis, urethral stricture, urethral diverticulum, glans dehiscence, breakdown, and cosmetic unfavorable outcome requiring redo-surgery. Complication rates depend on many factors which are not subject of this article. Reporting complications also depend on different factors. A recent survey of North American pediatric urologists clearly showed that there is a discrepancy between complication rates reported in the literature and the participants’ operative outcomes, regardless of practice setting, operative volume, or time in practice. The reasons for this interesting finding remain unclear (6). However, in an era of economic restraints and academic pressure, publication bias may be a significant factor in reporting complication rates. In a recent international hypospadias survey with nearly 500 participating pediatric urologists and pediatric surgeons, we sought to determine the strategies of follow-up and assessment of outcome (7). It was highly interesting that nearly 60% of all participants have a follow-up period of less than 6 months. On the other hand, only 10% of the surgeons would follow-up their patients until and beyond puberty (unpublished data). However, another survey performed at the 2011 ISHID meeting showed that more than 50% of the participants would follow their patients until puberty or beyond into adulthood. Table 1 shows randomly selected recent retrospective case series from 2013 (as sorted in PubMed by Recently Added). The range of follow-up lies between 6 weeks and 9 years. The majority of papers do not address the issue of “lost to follow-up” or “excluded from the study.” It has to be assumed that the follow-up rate usually is 100%. It has been criticized that follow-up periods – especially in Northern America – are short, perhaps too short to draw proper conclusions on outcome and complications (8). On the other hand, some believe that most of the complications appear within a short period post-operatively. Therefore, follow-up for 6 months or so appears to be sufficient (9). However, data from Gent show that there is a good long-term outcome without further complications in 75% of the patients. Among the 25% of patients who needed reoperation, only 47.37% appeared in the first year after surgery indicating the need for long-term follow-up (10). Moreover, growing and disturbing literature from adult urologists show the limitations of pediatric urologists’ view. There is an apprehension that pediatric urologists simply do not have enough epidemiological data on the incidence of failed hypospadias repair in childhood and currently there is no reliable estimation of the number of patients undergoing further surgery in adulthood or redo-surgery (11–13).

**Table 1:** **Follow-up period and percentage of lost to follow-up in randomly selected recent retrospective case series from 2013 (as sorted in PubMed by Recently Added)**.

Reference	Complication rate (%)	Follow-up period (months)	Lost to follow-up	Topic
Xu et al. (14)	18.1/21.5	22 (12–48)	Not given, obviously 100%	TIP vs. island flap
Vepakomma et al. (15)	45.8	6–35	Not given, obviously 100%	Modified Koyangi
Hadidi (16)	7	48 (12–108)	Not given, obviously 100%	Mathieu redo
Aslam et al. (17)	7	56 (3–103)	5%	TIP
Kallampallil and Hennayake (18)	4.3 (urethra), 18 non-retractile foreskin	27 (13–52)	5%	Foreskin reconstruction
Mane et al. (19)	12	32 (12–60)	Not given, obviously 100%	Modified TIP
Safwat et al. (20)	28.5	52 (3–102)	Not given, obviously 100%	Redo-surgery
Snodgrass et al. (21)	0–17	21 (1.5–82)	4%	TIP
Chandrasekharam (22)	12	22 (1–62)	Unclear	Island flap
El Dahshoury et al. (23)	3.3	27.6 (27–30)	100% follow-up	Island flap (double faced)
Dutta (24)	0	16 (8–38)	Not given, obviously 100%	Meatal and corpus spongiosum advancement

## Assessment of Cosmesis

Usually, cosmetic appearance is assessed by the surgeon. This is thought to be prone to bias, inaccuracy, and subjectiveness. Asking the parents or the patient (Are you satisfied about the cosmetic outcome? How is the urinary stream? Is the penis straight?) seems also not to be the most objective way to assess critical data. Hadidi proposed a score/assessment sheet including cosmetic and functional outcome and complications (25). It includes size of glans, size/appearance/location of meatus, curvature, complications (fistula, diverticulum, stricture), foreskin appearance, and functional outcome (urinary stream, erection). It is easy to apply, can be kept in the patient’s notes, and allows simple retrospective statistical evaluation. However, evaluation is still surgeon dependent. Mureau et al. were one of the first to apply a standardized approach to evaluate patient and surgeon satisfaction with the cosmetic surgical result, and the relation between penile length, meatal position, and patient satisfaction using a genital perception questionnaire for hypospadias patients. Not surprisingly, there was hardly any agreement between patient and surgeon satisfaction with patient penile appearance (26). Holland et al. then introduced the hypospadias objective scoring evaluation (HOSE) system where pediatric surgeons, a nurse, and one of the child’s parents independently assessed each patient. They showed that there was little inter-observer variation. The concept still seems very promising (27). There have been refinements like using digital photography with macro mode in a standardized fashion and with more external expertise in judging outcome. The assessment of cosmesis in hypospadias surgery was thought to be more objective when several health professionals, not involved in the surgery, compared the various methods of repair (28, 29). The most recent attempt for objective assessment of postoperative outcome is the Pediatric Penile Perception Score (PPPS), which seems to be the most reliable instrument to assess penile self-perception in children after hypospadias repair and for appraisal of the surgical result by parents and uninvolved urologists. The score includes size of penis, glans appearance, appearance of the meatus, penile skin, curvature, etc. rated by patient, parents, and surgeon (30). The PPPS has been validated for pediatric population as well as for adults (then called Penile Perception Score, PPS) (31). The Hypospadias Objective Penile Evaluation Score (HOPE) introduced by a national study group from the Netherlands established objectivity by using standardized photographs, anonymously coded patients, and independent assessment by a panel. They used reference pictures for meatal position and appearance, foreskin, general cosmesis, etc. Statistically, they reached a high intra- and inter-observer reliability, validity, and last but not least a high degree of reproducibility (32). However, there is still debate on what is most reliable and valid way to assess outcome (33, 34). Moreover, in most scores the preoperative findings and severity of hypospadias are not taken into account in assessing the final result. A recently developed preoperative Glans-Meatus-Shaft Score (GMS) seems to provide a brief and exact method with a good inter-observer reliability for describing the severity of hypospadias. Additionally, the GMS score appears to correlate with surgical outcome. The score assesses size of the glans, quality of the urethral plate, meatal position, and degree of chordee (35).

From the practical point of view, it is highly recommended to use standardized assessment tools for comparability and reproducibility, and to build up a prospective database. This can be facilitated as an institutional database, or even more favorable, in a multicenter international standardized database like I-DSD as shown later. Table 2 shows recent assessment tools and their pros and cons.

**Table 2:** **Recent hypospadias assessment tools and their pros and cons**.

Score	Items	Advantages	Disadvantages
HOSE (27)	Meatal location	Inter-observer reliability tested	Limited items
	Meatal shape		No general appearance
	Urinary stream		No penis size
	Erection/curvature		No adequate preoperative assessment
	Fistula	
Mureau (26)	Flaccid penile size	Assessment of penile size Not validated	Not tested for reliability and validity No erection/curvature Surgically non-correctable items No adequate preoperative assessment
	Penile thickness	
	Glandular size		
	Glandular shape	
	Position of meatus		
	Scars	
	Scrotum/testis		
	General appearance	
PPPS	Length of penis	High inter-rater reliability	Inherent subjective assessment
	Position and shape of meatus	Validated for surgeon and patient	No adequate preoperative assessment
	Glandular shape	
	Erection/curvature	
	General appearance	
HOPE	Position of meatus	Reference picture Implemented into prospective national database	Time consuming No adequate preoperative assessment
	Meatal shape	
	Shape of glans	
	Shape of skin		
	Penile torsion	
	Erection/curvature	
Hadidi score	Cosmesis and function	Easy to apply	Not validated and prone to subjectiveness

## Functional Outcome

Assessment of functional outcome in non-toilet trained boys is difficult. Functional outcomes are just beginning to be reported in the literature. Besides asking the patient about micturition, urinary flow rates after surgery in older patients have been first reported in 1970s (36). Weak flow rates have been contributed to real stenosis, low vesical pressure, rigidity and low compliance of the neourethra, pseudo-obstruction, and a lack of a natural corpus spongiosum (36). However, these explanations lack supporting evidence. Uroflow data include flow curve shape, maximum flow, micturition volume and post-void residual, and comparison to age-related flow rate nomograms, preferably as defined by the International Children’s Continence Society (ICCS) (37). Moreover, it has been well noted that boys with hypospadias show abnormal (though subclinical) flow patterns before and after surgery (38). Many studies support the importance of postoperative uroflow studies (39–42). Some studies show an improving tendency over time. Moreover, some note a weak correlation between flow and clinical symptoms. A recent systematic review recommends a uroflow study after toilet training. Children with obstructed flow parameters or borderline flows should be followed until adulthood. However, until long-term follow-up studies clarify the significance of abnormal flow parameters the significance of these studies remain uncertain (43). Interestingly, neither primary location of the meatus or surgical technique predicts poorer urinary function. However, there seems to be a correlation between severity of chordee and voiding function (41). On the other hand, a recent study describes functional obstruction of the neourethra following TIP defined as persistent obstructive voiding signs and symptoms in spite of apparently successful calibration or dilatation (16). Clinically obvious symptoms like a poor urinary stream, dribbling, incontinence, spraying, or hesitancy may be picked up easily. On the other hand, any subclinical lower urinary tract symptoms, primary or secondary bladder dysfunction, or overactive bladder are difficult to diagnose. These symptoms have been studied by invasive urodynamic studies and overactive is an accompanying entity in hypospadias (44). However, there is no place for routine urodynamic studies in the assessment of hypospadias. Last but not least, it has to be noted that uroflow studies in small children is very time consuming and can be somewhat frustrating. Although there are no large prospective studies, ultrasound with measurement of post micturition volume may offer another interesting non-invasive technique for postoperative assessment.

## Sexual Function, Quality of Life, and Psychosexual Life

Sexual behavior and sexual function after surgery in young adults are delicate topics and very demanding to assess. There are some studies assessing long-term psychosexual adjustment and sexual function matched with control groups including strength of libido, strength and duration of erection, penile appearance, penile size, curvature, problems with ejaculation (spraying, dribbling, retrograde ejaculation, premature ejaculation), masturbation activity, sexual activity, problems with intercourse, number of sexual partners, intimate relationships, and satisfaction with sexual life in general. These data show that patients with previous hypospadias surgery in general have rather good sexual function. However, there are differences in certain aspects of sexual behavior between patients with hypospadias and controls. Patients who had been operated for hypospadias are concerned about penile appearance. Particularly, penile size can obviously impact satisfaction (as in normal population). The more severe the hypospadias, the more dissatisfactory the long-term outcome and better cosmetic outcome is related to better sexual outcome. Recent data show a relatively high incidence of erectile dysfunction and premature ejaculation (45–48). A Swiss study showed a lower self-reported health-related quality (HRQ) of life in boys and adolescents following hypospadias repair related to penile self-perception fear of being ridiculed etc (49). Another recent Swiss study comparing adults who had hypospadias repair in childhood with a control group of circumcised men suggested that the HRQ is quite similar. However, poor genital self-perception again is correlated with an impaired mental HRQ (50). A case–control study from China showed that the incidence of anxiety and depression was significantly higher in adults following hypospadias repair. There was a correlation between the severity of symptoms and age at operation and penile size (51). Another Chinese study clearly showed that penile appearance and size of the penis have a major impact on psychosexual health (45). A small but promising study with adolescents following hypospadias repair showed that although there is impairment of body image and genital perception, the overall social, psychosocial, and sexual development seems to be normal (52). A systematic review from 2008 including only 13 studies with inconsistent quality showed that boys with hypospadias suffer from negative genital appraisal and sexual inhibitions. Psychological factors remain unclear (53). Surgery in the future will have to take much more into consideration the long-term consequences of esthetic and functional penile reconstruction in early childhood and how it will affect the patient in his later life physically, mentally, and emotionally.

A recent systematic review by Rynja et al. showed that there is a substantial lack in cosmetic, functional, and psychological long-term data. Moreover, quality of data is corrupted by low follow-up rate, heterogeneous patients and data, and a lack of validated questionnaires and control groups (54). Table 3 shows a number of parameters of follow-up which could be evaluated and surveyed in prospective long-term studies. Most surgeons would agree that the patient routinely should be seen within the first year of operation to assess short term outcome and to pick up complications. Voiding preferably is assed after toilet training. Yearly follow-up is desirable but extremely difficult to maintain. However, it is strongly recommended that the patient is seen after puberty (penile growth), as adolescent and sexually active man. It is a long way to go. However, there are promising studies coming up, e.g., the web-based prospective multicenter study by the Dutch Hypospadias Study Group. Another prospective multicenter online database will be installed in the I-DSD registry (www.i-dsd.org). The I-DSD registry is run by the I-DSD network which is a 5-year Medical Research Council funded initiative to support the development of an International DSD registry and network of clinical and research partners. The registry provides a means of connecting clinical and research centers around the world within a virtual environment and allows these experts to enter standardized information that will improve clinical practice, research, and understanding of these challenging conditions (55). Currently, a module for preoperative and postoperative assessment of hypospadias with the possibility of a prospective long-term follow-up regime is under development. International hypospadias surgeons will be invited to join the I-DSD registry and register their patients prospectively.

**Table 3:** **Follow-up parameters after hypospadias surgery**.

Parameter	Measurement	Items	Age and setting
History	Questionnaire	Age of operation	Any age
	Patient notes	Type of operation	
		Complications	
Voiding	Questionnaire	Satisfaction with voiding	After toilet training
		Stream	
		Spraying and Straining	
		Stand/sit	
		Post-void dribbling	
		LUTS	
	Uroflow	Volume	After toilet training (when symptoms?)
		*Q*_max_	
	Ultrasound	Residual volume	Adolescence
		Prostate	Adulthood
			Benefit not clear
	Score	International Prostate Symptom Score	Adulthood
		Expanded prostate index composite	
Cosmesis	Questionnaire Physical examination	Concern about abnormal appearance	Any time, particularly in sexually active patients
		Satisfaction with result	
		Penis size	
		Ashamed/fear of undressing	
		Being ridiculed	
		Curvature	
	Score	Junior Genital Perception Scale	Any time
		HOSE	
		PPPS	
		HOPE	
Sexuality	Questionnaire	Satisfaction with sexual function	In sexually active patients
		Masturbation	
		Intercourse	
		Erectile dysfunction	
		Ejaculatory problems	
		Inhibition in sexual contact	
		Relationship	
	Score	International index of erectile function	In sexually active patients
		Sexual Summary Score	
		Expanded prostate index composite	
Psychology	Questionnaire	Beck Depression Inventory	School age, adolescence and adulthood, involvement of clinical psychologist mandatory
		Goldberg General Health Questionnaire	
		Pediatric Quality of Life Inventory	
		Spielberger State-Trait Anxiety Questionnaire	
		Minnesota Multiphasic Personality Inventory	
		Child behavior checklist	
		Youth self report	
		Self-perception profile for adolescents	
		Case Western Reserve University Function Questionnaire	
		Self-Esteem and Relationship Questionnaire	

## Conclusion

Follow-up and adequate counseling of hypospadias patients up to adult life is necessary, although demanding. Long-term assessment should be designed in prospective studies. Somehow in the future, we will be able to give an accurate estimation of the long-term consequences of being born with hypospadias.

## Conflict of Interest Statement

The author declares that the research was conducted in the absence of any commercial or financial relationships that could be construed as a potential conflict of interest.
